# Fighting Cancer Stem Cell Fate by Targeting LIS1 a WD40 Repeat Protein

**DOI:** 10.3389/fonc.2019.01142

**Published:** 2019-10-31

**Authors:** Felix M. Brehar, Mihnea P. Dragomir, George E. D. Petrescu, Radu M. Gorgan

**Affiliations:** ^1^Department of Neurosurgery, “Carol Davila” University of Medicine and Pharmacy, Bucharest, Romania; ^2^Department of Neurosurgery, “Bagdasar-Arseni” Clinical Emergency Hospital, Bucharest, Romania; ^3^Department of Surgery, “Carol Davila” University of Medicine and Pharmacy, Bucharest, Romania; ^4^Department of Experimental Therapeutics, The University of Texas MD Anderson Cancer Center, Houston, TX, United States

**Keywords:** cancer stem cell, Lis1, chemoresistance, radioresistance, WD40 protein

## Abstract

Cancer is one of the most frequent and devastating diseases. Previous reports have shown that radio and chemo-resistant cancer stem cell (CSC) population is primarily responsible for cancer recurrences after radiotherapy and chemotherapy. Other studies demonstrated that Lissencephaly-1 (LIS1) protein, also known as platelet activating factor acetylhydrolase 1b regulatory subunit 1 (PAFAH1B1), a dynein-binding protein involved in neural stem cell division, plays a crucial role in maintaining CSC population in hematological malignancies. Moreover, one recent report demonstrated that *LIS1* gene is preferentially expressed in CD133+ glioblastoma cells and may have also an important role in regulating CD133+ CSC in glioblastoma. The hypothesis of this paper is that LIS1 plays a key role in maintaining CD133+ CSC population in various solid cancers by orientating the cell division plane through an interaction with dynein and therefore controlling the stem cell fate regulatory mechanism. As CD133+ CSC population is responsible for radio- and chemo-resistance, which finally determines the cancer recurrences and metastases, identifying the molecular mechanisms which regulate the CD133+ CSC population represents a major target for cancer research. Given the structure of LIS1, which contains WD40 repeat domain, small peptide inhibitors could be used to alter its function. Therefore, the impact of confirming this hypothesis is significant because *LIS1* may become an important molecular target for future adjuvant anticancer therapies directed against radio- and chemo-resistant CSC population.

## Introduction

Cancer is one of the most frequent and devastating diseases. Death occurs in most cases due to systemic progression of the primary tumor after surgical and oncologic treatment (radiotherapy and chemotherapy). Recent studies have shown that cancer recurrences after treatment are primarily due to a radio- and chemo-resistant cancer stem cell (CSC) population (1–3). Therefore, one of the major goals of modern cancer research is to develop a therapy against CSC population. New evidence suggests that the self-renewal of CSC in hematological malignancies is dependent on a molecular mechanism controlled by LIS1 protein (4) and additionally, some polymorphic variants of the *LIS1* gene have an increased risk for acute myeloid leukemia (5). LIS1 is a protein which also plays a crucial role during early stages of brain development (6–9). This protein, which interacts with dynein, influences the orientation of the division plane relative to the cell determinants segregation and thus it has an essential role in controlling the stem cell fate (10–12). Our recent studies demonstrate by using qRT-PCR that although no statistically significant difference exists between the mRNA expression level of *LIS1* gene in glioblastoma compared with normal brain tissue, a spectacular difference (expression of up to 60-fold higher) was found between *LIS1* mRNA expression in CD133+ compared to CD133- primary glioblastoma cells (13, 14). Also, previous work proved the important role of LIS1 in maintaining CD133+ cell populations in glioblastoma, as well as the involvement of LIS1 in adhesion, migration and proliferation of CD133+ U87 glioblastoma cell line. Silencing *LIS1* gene in U87 demonstrated a decrease of up to 6-folds of the percentage of CD133+ glioblastoma cells and a reduction of cell proliferation, migration and adhesion (13, 14). The shLIS1-U87 cells were significantly more sensitive to radiation (due to decreased radio-resistant CD133+ cell population) as compared to control U87 (14). Also, recent results showed that shLIS1 U87 cells treated with temozolomide and radiotherapy have a significant lower proliferation rate than control U87 cells (15). It is notable that although there is a controversy regarding the value of CD133+ as a CSC marker, we demonstrated that in glioblastoma, *LIS1* (also a marker of stemness) expression is up-regulated in CD133+ cells, thus suggesting, along with other authors, that CD133 maintains its value as reliable marker of CSC (16). Additional papers reported that in other common types of solid cancer, CD133+ cells have an important role in oncogenesis, tumor recurrence and radio- and chemo-resistance. Specifically, CD133+ cells are responsible for the highly aggressive behavior of triple-negative breast cancer (a form virtually unresponsive to standard oncological treatment) and small cell lung cancer (17, 18). Also, previous studies revealed an important role of CD133+ tumor cells in oncogenesis and development of chemo- and radio-resistance in melanoma (19) and glioblastoma (2). All these studies, unequivocally, demonstrated that CD133+ cancer cells are resistant to radio- and chemotherapy, but the mechanism of maintaining the stem like properties are not evaluated. Herein, we propose *LIS1* as an essential gene in maintaining the CD133+ cancer cell population in solid malignancies and targeting LIS1 could improve the efficiency of standard anticancer therapies.

## Proposed Role of LIS1 in Regulating Cancer Stem Cells

**Figure 1:**
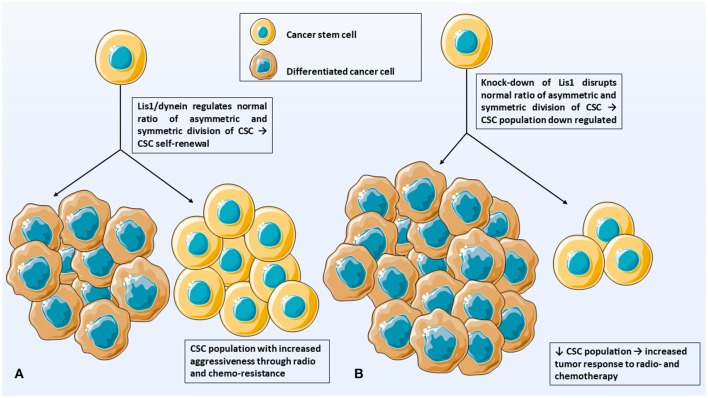
Illustrates the hypothesis of this paper. **(A)** LIS1/dynein interaction plays a crucial role in maintaining the CD133+ CSC population in solid tumors. **(B)** Silencing *LIS1* gene will shut-down LIS1-dynein interaction and deregulate normal ratio of asymmetric and symmetric divisions. CSC population is down-regulated which increase tumor response to radio- and chemotherapy. CSC, cancer stem cells; DCC, differentiated cancer cells.

This paper hypothesizes that *LIS1* plays a key role in maintaining CD133+ cancer stem cells population in solid cancers. One possible mechanism through which LIS1 maintains CD133+ CSC population could be the stem cell fate controlling mechanism. Zimdahl et al. already demonstrated that this mechanism is involved in the pathogenesis of hematopoietic malignancies (4) and we hypothesize that it might also have a role in solid cancers. This molecular mechanism uses LIS1-dynein interaction to influence the cell determinants segregation and the ratio of asymmetric/symmetric cell divisions (Figure 1A). According to this study, silencing *LIS1* gene will deregulate the normal ratio of asymmetric and symmetric division of CSC and down-regulate the CSC population (Figure 1B). As CSC population is primarily responsible for tumor radio- and chemo-resistance, the final result will be an increasing of tumor response to radio- and chemotherapy (Figure 1B).

However, this molecular mechanism of stem cell fate control may not be only LIS1-dependent, other mechanisms involved in maintaining CSC population exist. Additionally, we previously demonstrated that silencing LIS1 alters also other functions of CD133+ glioblastoma cells, like cell proliferation, adhesion and migration (13). These cell functions involve active reorganization of the microtubules which requires a proper function of LIS1. Therefore, silencing LIS1 will not deregulate only the formation of new CSCs but will interfere with the proliferation and migration of newly formed CSCs. The final result will be a progressive decreasing of CSC population with an increased response to standard anticancer therapy.

Some authors report that *LIS1* is down-regulated in hepatocellular carcinoma and that *lis1* knock-out in mice models promotes tumorigenesis (20, 21). Our previous results show that the levels of *LIS1* are low in the whole population of glioblastoma cells, the difference residing in the levels of *LIS1* in glioblastoma CSC, which are upregulated (14). Therefore, the levels of *LIS1* in hepatocellular carcinoma CSC should be measured since this subpopulation might be responsible for the tumor progression.

**Figure 2:**
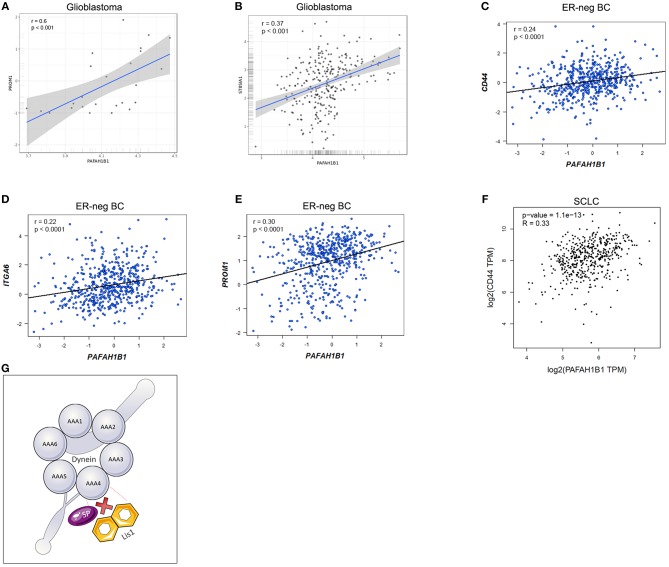
**(A)** Depicts the positive correlation between *LIS1* and *PROM1* (encoding CD133) in glioblastoma microvascular proliferation samples; **(B)** represents the positive correlation between *LIS1* and *ST8SIA1*(encoding A2B5 marker) in glioblastoma samples; **(C–E)** shows the positive correlation between *LIS1* and the CSC markers for estrogen-receptor negative breast cancer (CD44+, CD49f+, and CD133+); **(F)** demonstrates the correlation between *LIS1* and CD44 in small cell lung cancer samples; **(G)** illustrates the hypothetical mechanism through which small peptide inhibitors could interfere with the dynein/LIS1 molecular interaction. LIS1 contains WD40 repeat domains and small peptide inhibitors could be used to compete for the binding sites and subsequently alter the function of LIS1. AAA1-AAA6, dynein domains; ER-neg BC, estrogen-receptor negative breast cancer; ITGA6, integrin subunit alpha 6 (CD49f); *PAFAH1B1*, platelet activating factor acetylhydrolase 1b regulatory subunit 1 (*LIS1*); SCLC, small cell lung cancer; SP, small peptide inhibitor.

In order to confirm the role of LIS1 in maintaining the CSC population in different tumor types, we checked for correlations between *LIS1* mRNA level and specific stem cell markers in multiple cancers. For example, we analyzed using the GlioVis Portal (22) the correlation between mRNA expression of *LIS1* and *PROM1* (CD133 encoding gene) in glioblastoma samples from the Ivy Glioblastoma Atlas Project (Ivy GAP) (23). Although there is no correlation between the mRNA levels of the two aforementioned genes in the overall glioblastoma samples, there is a highly significant positive correlation (*r* = 0.6; *p* < 0.001) in the microvascular proliferation sample subgroup (Figure 2A). This can be explained through the affinity of stem cells to the vascular niche (24–27). Moreover, *LIS1* correlates with the *ST8SIA1* gene responsible for encoding A2B5 protein, which is also a CSC marker in glioblastoma (28, 29) (Figure 2B). Also, we analyzed for estrogen receptor negative (ER-negative) breast cancer data from multiple databases (TCGA, GSE81538, GSE96058) (30, 31) using the Breast Cancer-GenExMiner Portal (32, 33). We observed that *LIS1* positively correlates with the ER-negative breast cancer CSC markers [i.e., CD133+, CD49f+, CD44+ (34)] (Figures 2C–E). Additionally, *LIS1* positively correlates with the CD44+ CSC marker for lung cancer (35), according to the GEPIA portal (36) which analyses data from TCGA small cell lung cancer samples (30) (Figure 2F). Regarding the role of LIS1 as a prognostic marker, Lo et al. reported that high levels of LIS1 protein expression in tumor samples from patients with lung adenocarcinoma are associated with a poor prognosis (37).

The universal role of *LIS1* gene in maintaining the stem cell population, both normal and cancerous is supported by the fact that *LIS1* is an evolutionary conserved gene (38). First, there has been demonstrated the role of LIS1 molecule in regulating the stem fate by influencing the segregation of the cell determinants (cellular fate) in *Drosophila* neuroblast (11). Then it has been shown the role of LIS1 in more complex organisms (mammals) where LIS1 is required for neuroepithelial stem cell proliferation (12). Another study demonstrated that LIS1 plays an essential role in maintaining both normal and malignant hematopoietic stem cells (4). A more recent report has shown that *LIS1* gene is preferentially expressed in CD133+ glioblastoma cells and may play an essential role in regulating CD133+ glioblastoma cells function (14). Therefore, in light of these previously published studies, we suggest that LIS1 may play a universal role in regulating the CD133+ cancer cell population in solid cancer.

LIS1 exerts its role by LIS1-dynein direct interaction which influences the orientation of mitotic spindle and cell determinants segregation (4). The underlying molecular mechanism of LIS1-dynein interaction has been previously reported by Huang et al. They demonstrated that LIS1 induce conformational changes when it attaches to dynein molecule and therefore may act as a “clutch” between ATP-ase and microtubule binding domains of this molecule (39). Moreover, four highly conserved amino acids which play a key role in LIS1-dynein interaction were identified: Lysine (2721), Aspartate (2725), Glutamate (2726 and 2727). Also, recent work suggests that lis1 can regulate dynein by binding it in either a weak or a tight manner, providing new insights for altering lis1 functions *in vivo* (40). However, this detailed molecular mechanism was demonstrated using purified proteins from *Saccharomyces cerevisiae*. As LIS1 is an evolutionary highly conserved molecule, we suggest that the molecular mechanism of LIS1-dynein interaction could be similar in mammals including *Homo sapiens*. Also, LIS1 contains one of the most encountered protein interactions domain in human, the WD40 repeat domain (41), incorporating in its structure seven WD40 repeats (38). Since WD40 repeat inhibitors are already being evaluated in clinical trials (42), we hypothesize that CD133+ CSC could be down-regulated by interfering with LIS1-dynein interaction using small peptide inhibitors (Figure 2G). The final result would be similar to silencing *LIS1* expression, namely a significantly decreased CSC population that would increase the tumor response to radio- and chemotherapy (Figure 1B).

## Future Directions

To verify this hypothesis, it is necessary to evaluate the expression and function of LIS1 in CD133+ CSC isolated from the most frequent solid cancers: breast cancer, lung cancer, colorectal cancer and melanoma.

Firstly, the expression of LIS1 in CD133+ CSC population isolated from tumor cell lines and primary cultures of breast cancer, lung cancer, melanoma, and colorectal cancer should be evaluated. This implies the isolation and characterization of CD133+ CSC populations using some of the most common tumor cell lines and primary cultures in the aforementioned types of cancer. The expected result is that the CD133+ tumor cells preferentially over-express LIS1 in a similar manner with CD133+ cells isolated from glioblastoma (14).

Secondly, the mechanistic function(s) of LIS1 in CD133+ CSC population isolated from cancer cell lines should be evaluated. In order to accomplish this, LIS1 shRNA knock-down or CRISPR-Cas9 knock-out *in vitro* for each of the four studied cancers will be achieved. To determine the impact of silencing/knocking-out *LIS1* in CD133+ tumor cell population, the percentage of CD133+ cells in cancer cell lines transfected or edited at the genome level will be measured using flow cytometry and western blotting and compared with controls. There will also be conducted *in vitro* functional studies and *in vivo* tumor growth and metastasis experiments in order to evaluate the *LIS1* gene silencing effect on proliferation, adhesion, migration and tumorigenesis of CD133+ CSC. The anticipated result will be that silencing *LIS1* influences the fate of CSC and therefore the consequent change in stem cell markers expression such as a decrease of the CD133+ CSC fraction (for other solid cancers we expect a decrease in other CSC markers such as CD44, CD49f, A2B5) in the silenced/knocked-out *LIS1* cancer cells, as well as lower proliferation, adhesion and migration rates compared with controls.

Thirdly, studies at the molecular level that would elucidate the mechanism(s) through which LIS1 regulates CSC population in solid cancers should be performed. The working hypothesis is that LIS1 induces the self-renewal of CSC by interacting with dynein protein. Through this interaction, the correct orientation of the division plane occurs and a correct ratio between asymmetric and symmetric cell divisions necessary for the maintenance of the CSC population is ensured. This mechanism has been demonstrated by Zimdahl et al. (4) for malignant hemopathies. As part of this objective is necessary to evaluate whether this mechanism is also present for CSC self-renewal in solid cancers. It has been reported that by mutating a series of well-conserved amino acids in dynein, the interaction between LIS1 and dynein is altered (39). Several other studies present evidence about the role of dynamitin (43), Rab6a (44), and PDE4 (45) in dissociating LIS1-dynein complex. Therefore, it is possible to induce mutations in dynein or to over-expression one of the negative regulators of the LIS1-dynein interaction and evaluate *in vitro* and *in vivo* if dissociating the LIS1-dynein complex will recapitulate the results of silencing LIS1 (e.g., decreasing the percentage of CD133+CSC cells, decreasing the proliferation, migration and tumorigenesis *in vitro* and decreasing the metastatic potential *in vivo* of CD133+ CSC cells).

Additionally, it is important to understand also the upstream mechanisms that induce the overexpression of LIS1. In recent years, there has been a growing interest in studying epigenetic mechanism that change gene expression in cancer, especially at post-transcriptional level. MicroRNAs (miRNAs) are a subtype of small non-coding RNAs with a role in regulating gene expression (46). The role of miRNAs in the diagnostic and their possible therapeutic actions in gliomas has been reviewed elsewhere (47). Yang et al. proved that the overexpression of miR-144 (that was found down-regulated in cholangiocarcinoma tissue samples) in cholangiocarcinoma cell lines is capable to inhibit their proliferation and invasion by targeting *LIS1* (48). Also, comparable results were obtained when LIS1 was knocked-down. Hence, this could be another possible mechanism through which LIS1 function could be altered, that requires further studying. Moreover, when developing an anti-LIS1 therapy, researchers should note that *LIS1* deletion in hematopoietic system was reported to lead to a significant decrease in blood cells, due to impaired hematopoietic stem cell function and consequently embryonic death (4). Therefore, it is important to target LIS1 specifically in the tumor cell population using a targeted/local delivery system.

## Conclusion

The impact of this hypothesis is particularly important because its major goal is to target the tumor cell population responsible for cancer recurrence and resistance to current oncological treatments (radio- and chemo-therapy). Compared to previous studies, which evaluate the role of Lis1 gene in leukemia or glioblastoma (4, 13, 14), this approach also covers other types of solid cancers, hence having a greater impact on our understanding of the molecular mechanisms of replicative immortality of cancers. Basically, a holistic approach is proposed, guided by the assumption that Lis1 dependent mechanism of maintaining the CSC population is universal for all types of cancer.

Besides the benefit of creating a new research opportunity in the field of CSC, this idea also has a significant social impact. It is well-known that the cancer patients represent at this moment one of the largest segments of chronic patients in the general population with a high rate of mortality and morbidity. The poor prognosis is primarily due to the resistance to current treatments (radio- and chemotherapy) and the tendency of the tumor to relapse. Given the fact that the CD133+ CSC population is the one responsible for radio- and chemo-resistance, it becomes evident that the development of a molecular therapy to target CSC tumor cells would have a major impact on the health condition of the population.

## Data Availability Statement

For gene expression we analyzed the following publicly available datasets: https://glioblastoma.alleninstitute.org/static/home; https://portal.gdc.cancer.gov; http://bcgenex.centregauducheau.fr.

## Author Contributions

FB came up with the hypothesis of the paper. FB, GP, MD, and RG wrote the manuscript and reviewed and approved the final form of the manuscript. GP, FB, and MD made the figures.

### Conflict of Interest

The authors declare that the research was conducted in the absence of any commercial or financial relationships that could be construed as a potential conflict of interest.
